# High-Intensity Exercise Training Protects the Brain Against Autoimmune Neuroinflammation: Regulation of Microglial Redox and Pro-inflammatory Functions

**DOI:** 10.3389/fncel.2021.640724

**Published:** 2021-02-23

**Authors:** Yifat Zaychik, Nina Fainstein, Olga Touloumi, Yehuda Goldberg, Liel Hamdi, Shir Segal, Hanan Nabat, Sofia Zoidou, Nikolaos Grigoriadis, Abram Katz, Tamir Ben-Hur, Ofira Einstein

**Affiliations:** ^1^Department of Physical Therapy, Faculty of Health Sciences, Ariel University, Ariel, Israel; ^2^Department of Neurology, The Agnes Ginges Center for Human Neurogenetics, Hadassah—Hebrew University Medical Center, Jerusalem, Israel; ^3^B’ Department of Neurology, AHEPA University Hospital of Thessaloniki, Thessaloniki, Greece; ^4^Åstrand Laboratory of Work Physiology, The Swedish School of Sport and Health Sciences, GIH, Stockholm, Sweden

**Keywords:** exercise training, autoimmunity, neuroprotection, microglia, multiple sclerosis, experimental autoimmune encephalomyelitis

## Abstract

**Background**: Exercise training induces beneficial effects on neurodegenerative diseases, and specifically on multiple sclerosis (MS) and it’s model experimental autoimmune encephalomyelitis (EAE). However, it is unclear whether exercise training exerts direct protective effects on the central nervous system (CNS), nor are the mechanisms of neuroprotection fully understood. In this study, we investigated the direct neuroprotective effects of high-intensity continuous training (HICT) against the development of autoimmune neuroinflammation and the role of resident microglia.

**Methods**: We used the transfer EAE model to examine the direct effects of training on the CNS. Healthy mice performed HICT by treadmill running, followed by injection of encephalitogenic proteolipid (PLP)-reactive T-cells to induce EAE. EAE severity was assessed clinically and pathologically. Brain microglia from sedentary (SED) and HICT healthy mice, as well as 5-days post EAE induction (before the onset of disease), were analyzed *ex vivo* for reactive oxygen species (ROS) and nitric oxide (NO) formation, mRNA expression of M1/M2 markers and neurotrophic factors, and secretion of cytokines and chemokines.

**Results**: Transfer of encephalitogenic T-cells into HICT mice resulted in milder EAE, compared to sedentary mice, as indicated by reduced clinical severity, attenuated T-cell, and neurotoxic macrophage/microglial infiltration, and reduced loss of myelin and axons. In healthy mice, HICT reduced the number of resident microglia without affecting their profile. Isolated microglia from HICT mice after transfer of encephalitogenic T-cells exhibited reduced ROS formation and released less IL-6 and monocyte chemoattractant protein (MCP) in response to PLP-stimulation.

**Conclusions**: These findings point to the critical role of training intensity in neuroprotection. HICT protects the CNS against autoimmune neuroinflammation by reducing microglial-derived ROS formation, neurotoxicity, and pro-inflammatory responses involved in the propagation of autoimmune neuroinflammation.

## Introduction

Recent evidence highlights a favorable outcome for extensive exercise training in the treatment of Parkinson’s disease (PD; Frazzitta et al., 2013; Petzinger et al., 2013), Alzheimer disease (AD; Radak et al., 2010; Hoffmann et al., 2016), stroke (Luo et al., 2020) and mood and anxiety disorders (Dunn et al., 2005). Even in the absence of pathological conditions, exercise training has been shown to improve cognitive function and spatial memory through increased regional neurogenesis and plasticity in mice (Li et al., 2013). Accordingly, there is a large body of evidence indicating beneficial outcomes of exercise among multiple sclerosis (MS) patients (Heine et al., 2016; Motl, 2020). Although the favorable effects of exercise on brain health are well accepted, a thorough understanding of the neuroprotective effects of exercise on autoimmune neuroinflammatory diseases is still lacking.

We recently demonstrated peripheral-systemic immunomodulatory effects of moderate-intensity continuous training (MICT) in transfer experimental autoimmune encephalomyelitis (EAE), an animal model used for the study of autoimmune-mediated disease of the central nervous system (CNS) (Einstein et al., 2018). MICT reduced the encephalitogenicity of autoreactive T cells and attenuated the clinical severity of transfer EAE. We further demonstrated that high-intensity continuous training (HICT) induced superior benefits in preventing systemic autoimmunity in EAE as compared to MICT (Fainstein et al., 2019). Interestingly, we showed that MICT did not result in a direct protective effect on the CNS from encephalitogenic T cells (Einstein et al., 2018). This prompted us to investigate whether HICT, in addition to its superior systemic immunomodulatory effect, will also induce a protective effect directly on the CNS against autoimmune neuroinflammation, as well as potential mechanisms whereby training may induce direct neuroprotection.

Accumulating data indicate that microglia are pivotal in mediating neuroinflammation, demyelination, and neurodegeneration in MS and EAE (Chu et al., 2018). Toxic activation of microglia is apparent during early and late MS and EAE and correlates with axon and oligodendrocyte pathology (Henderson et al., 2009). Microglial modulation is considered to promote CNS well-being by exercise training (Mee-Inta et al., 2019). These findings prompted us to examine whether microglia play a role in training-mediated neuroprotection.

Here, we investigate the direct effects of HICT on neuroprotection and the development of autoimmune neuroinflammation in EAE, employing the transfer EAE model, whereby we administer encephalitogenic T cells into HICT and sedentary (SED) mice. We show that HICT protects the brain from encephalitogenic T cells, resulting in reduced neuroinflammation and tissue injury. HICT results in reduced toxic and pro-inflammatory activation of resident microglia.

## Materials and Methods

### Experimental Animals

Female SJL/JCrHsd mice (6–7 weeks of age) were purchased from Envigo Inc., Israel. Animal experimentation was approved by the Institutional Animal Care and Use Committee (approval number IL-114-08-16). The studies were conducted following the United States Public Health Service’s Policy on Humane Care and Use of Laboratory Animals.

### Experimental Design

**Figure 1:**
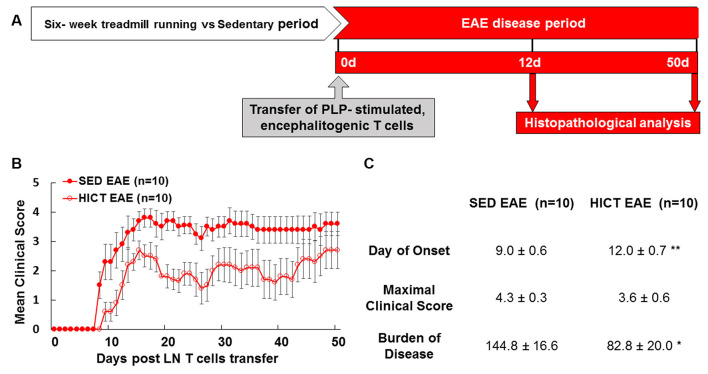
High-intensity training attenuates the clinical course of recipient mice in a transfer model of experimental autoimmune encephalomyelitis (EAE). **(A)** Experimental timeline to investigate the direct effects of exercise training on the central nervous system (CNS) in the transfer EAE model. Healthy mice were subjected to a 6 week-high-intensity continuous training (HICT) treadmill-running program and served as recipient mice to further develop EAE. Another group of naïve donor mice was immunized with proteolipid (PLP) peptide, and after 10 days their lymph node (LN)-T cells were removed and stimulated in culture with PLP peptide. The encephalitogenic T cells were injected into either HICT, 72 h before the last exercise bout, or sedentary (SED) control recipient mice, which developed EAE and were scored daily for neurological symptoms up to 50 days post-transfer. Mice were sacrificed for central nervous system histopathology analyses at 12 days and 50 days post EAE induction, at the acute phase and chronic phase of the disease, respectively. Clinical course **(B)** and clinical parameters **(C)** of EAE in recipient sedentary (SED EAE, *n* = 10) and HICT (HICT EAE, *n* = 10) mice following the transfer of encephalitogenic T cells. The severity of EAE was scored on a 0–6 scale. Transfer of encephalitogenic T cells to HICT recipients induced a significantly milder EAE course. DOO, day of onset; MCS, maximal clinical score; BOD, burden of disease. Data are mean ± SE. *p < 0.05, **p < 0.01 vs. SED EAE.

The proteolipid (PLP) 139–151 transfer EAE model in mice was utilized as previously described (Einstein et al., 2007, 2018; Fainstein et al., 2019). This model enables isolation of the direct effects of HICT on the CNS, as indicated by induction of EAE following the transfer of encephalitogenic T cells (Figure 1A). Healthy mice were subjected to a 6-week treadmill high-intensity continuous training (HICT) program. PLP-reactive, encephalitogenic lymph node (LN)-T cells from donor mice were injected into trained mice, 72 h before the last exercise bout. Sedentary (SED) mice, that were kept under identical environmental conditions as the trained group, but did not perform treadmill running during the experimental period, were injected with the same PLP-reactive encephalitogenic T cells and served as controls. We examined whether the treadmill running program of the recipient mice before the transfer of encephalitogenic T cells induced direct neuroprotective effects on the CNS as follows: (1) by *in vivo* clinical and pathological severity of EAE following the transfer of encephalitogenic T cells (Figure 1A); and (2) by *ex vivo* functional analysis of resident microglia isolated from HICT and SED mice at two time-points: from healthy mice, at the end of the training program or sedentary period, before the transfer of encephalitogenic T cells (Figure 5A) and from HICT and SED mice 5 days after the transfer of encephalitogenic T cells and EAE induction (pre-onset EAE, Figure 6A). Infiltration of systemic immune cells into the CNS and clinical onset of EAE occurred after 5 days following the transfer of encephalitogenic T cells.

**Figure 2:**
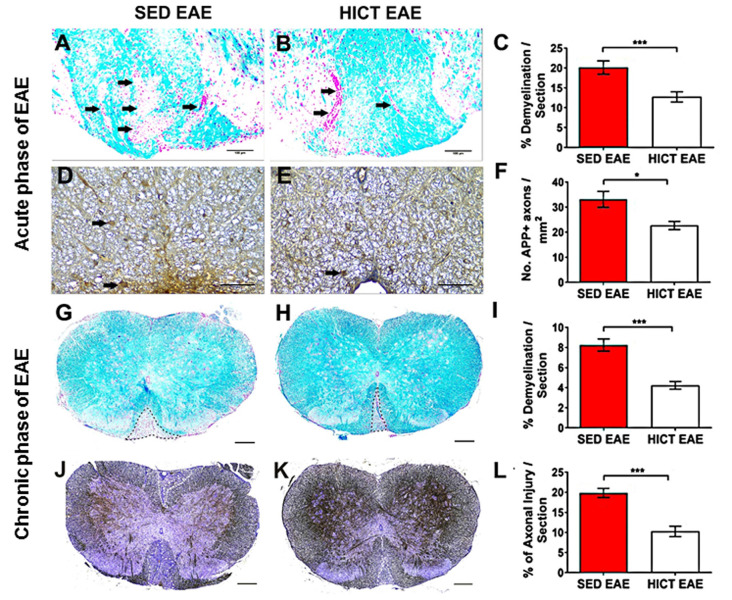
Reduction of tissue pathology in the spinal cords of high-intensity trained EAE mice. Evaluation of demyelination **(A–C,G–I)** and axonal damage **(D–F,J–L)** on cross-sections of the spinal cords of sedentary (SED EAE: **A,D,G,J**, *n* = 6) and high-intensity continuous trained (HICT EAE: **B,E,H,K**, *n* = 6) mice that were injected with encephalitogenic T cells and developed EAE. **(A–F)** Twelve days post EAE induction, acute phase of the disease; **(G–L)** 50 days post EAE induction, chronic phase of the disease. **(A,B)** Arrows indicate areas of demyelination; **(D,E)** arrows indicate amyloid precursor protein APP + injured axons; **(G,H)** dashed lines indicate area of demyelination; **(J–K)** Dashed lines indicate the area of axonal loss. **(C,F,I,L)** Quantification of tissue pathology in spinal cord white matter. Luxol fast blue (LFB) histochemistry with periodic acid Schiff (PAS) counterstaining showed a reduction in the area of demyelination in HICT EAE **(B,H)** vs. SED EAE **(A,G)** at the acute **(C)** and chronic **(I)** phases of the disease. In HICT EAE there were fewer APP + injured axons (**E**, arrows) than in SED EAE **(D)** at the acute phase **(F)**. Bielschowsky staining at the chronic phase of disease showed less axonal damage and axonal loss in HICT EAE mice **(K)** than in control SED EAE mice **(J,L)**. Scale bars: **(A,B,G–K)** = 100 μm; **(D–E)** = 50 μm; Data are mean ± SE. *p < 0.05, ***p < 0.001.

**Figure 3:**
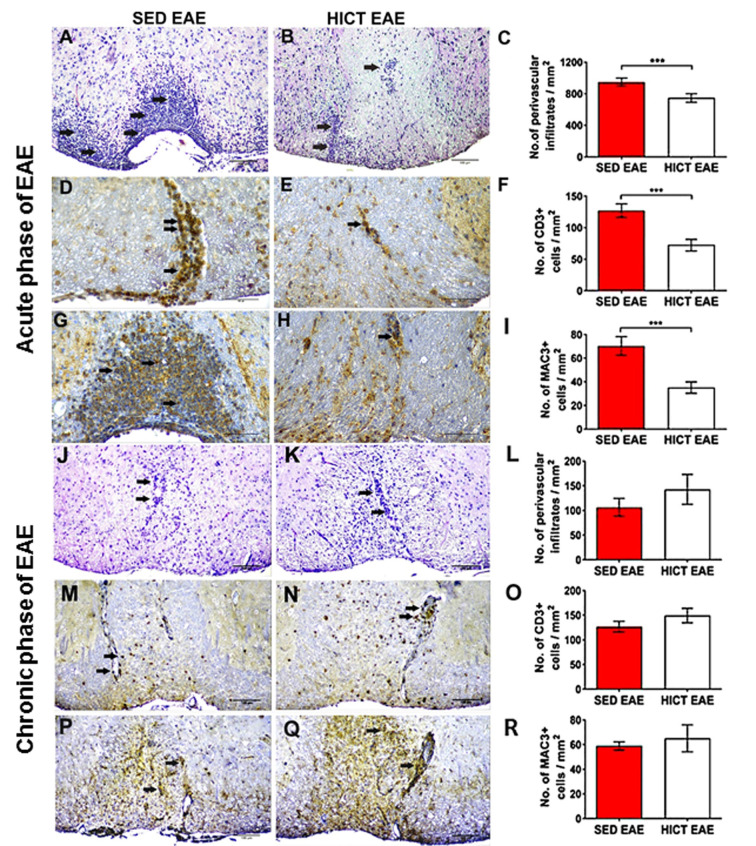
Reduction of acute autoimmune inflammation in the spinal cords of high-intensity trained EAE mice. Immunohistochemistry for inflammatory infiltrates **(A–C,J–L)**, CD3 T cells **(D–F,M–O)**, and Mac3 macrophages **(G–I,P–R)** on cross-sections of the spinal cords of sedentary (SED EAE: **A,D,G,J,M,P**, *n* = 6) and high-intensity continuous trained (HICT EAE: **B,E,H,K,N,Q**, *n* = 6) mice that were injected with encephalitogenic T cells and developed EAE. **(A–I)** Twelve days post EAE induction, acute phase of the disease, **(J–R)** 50 days post EAE induction, chronic phase of the disease. **(A,B,J,K)** Arrows indicate perivascular infiltrates; **(D,E,M,N)** arrows indicate perivascular CD3+ T cells; **(G,H,P,Q)** arrows indicate Mac3+ macrophages. **(C,F,I,L,O,R)** Counts of inflammatory cell types in spinal cord white matter. At the acute phase of the disease, in HICT EAE mice there was a significant reduction in total perivascular immune cell infiltrations **(B,C)**, in CD3+ T cells **(E,F)** and Mac3+ macrophages **(H,I)** vs. SED EAE mice (**A,D,G**, respectively). Similar numbers of perivascular immune cell infiltrations **(L)**, CD3+ T cells **(O)**, and Mac3+ macrophages **(R)** were counted in SED EAE **(J,M,P)** and HICT EAE (**K,N,Q**, respectively) mice at the chronic phase of the disease. Scale bars: **(A,B,J–Q)** = 100 μm, **(D–H)** = 50 μm. Data are mean ± SE. ***p < 0.001.

**Figure 4:**
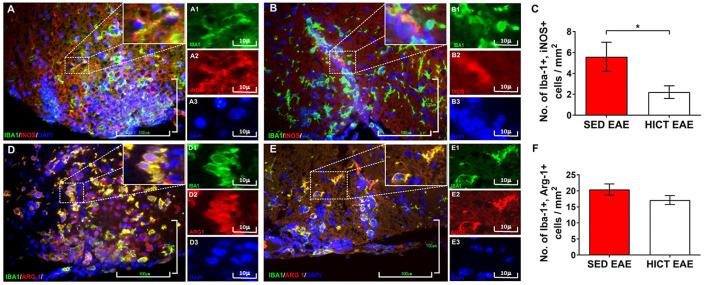
Reduction of neurotoxic microglia in the spinal cords of high-intensity trained EAE mice. Double immunofluorescent stainings for Iba-1 and inducible nitric oxide synthase (iNOS) M1 phenotype microglia **(A,B)** and Iba-1 and arginase-1 (Arg-1) M2 phenotype microglia **(C,D)** on cross-sections of the spinal cords of sedentary (SED EAE: **A,A1–3,D,D1–3**; *n* = 6) and high-intensity continuous trained (HICT EAE: **B,B1–B3,E,E1–3**; *n* = 6) mice at 12 days post EAE induction by injection with encephalitogenic T cells (acute phase). **(C,F)** Counts of double-positive cells in spinal cord white matter. In HICT EAE mice, there was a significant reduction in double-positive Iba-1+, iNOS+ M1 type toxic microglia **(B)** vs. SED EAE mice **(A,C)**. Similar numbers of double-positive Iba-1+, Arg-1+ M2 type microglia were counted in SED EAE **(D)** and HICT EAE **(E,F)**. Iba-1: **(A,A1,B,B1,D,D1,E,E1)**—green; iNOS: **(A,A2,B,B2)**—red; Arg-1: **(D,D2,E,E2)**—red; nuclear staining with DAPI: **(A,A3,B,B3,D,D3,E,E3)**—blue. Scale bars: **(A,B,D,E)** = 100 μm; **(A1–3,B1–3,D1–3,E1–3)** = 10 μm. Data are mean ± SE. *p < 0.05.

**Figure 5:**
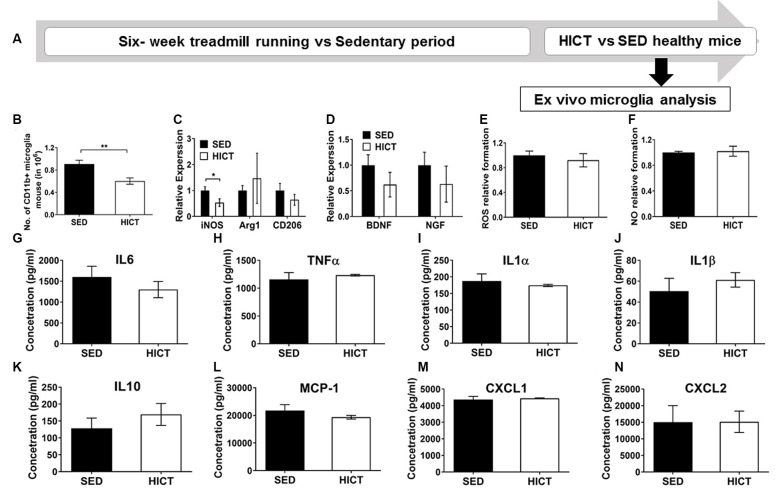
Decreased levels but preserved function of brain microglia in healthy high-intensity trained mice. **(A)** CD11b+ microglia were isolated from the brain of mice following 6 weeks of high-intensity continuous training (HICT), 48 h after the last exercise bout, or sedentary period (SED) and analyzed *ex vivo* on day of isolation **(B–D)** or after additional 24 h in culture with lipopolysaccharide (LPS) stimulation **(E–N)**. **(B)** Number of CD11b+ microglia per mouse at the day of microglia isolation (*n* = 8/group). mRNA levels of inducible nitric oxidase synthase (iNOS), arginase (Arg)-1 and CD206 M1/M2 phenotype markers **(C)** and brain-derived neurotrophic factor (BDNF) and nerve growth factor (NGF, **D**) in CD11b+ microglia at the day of isolation (*n* = 5/group). Levels of reactive oxygen species (ROS, **E**) and nitric oxide (NO, **F**) in supernatants of LPS-stimulated CD11b+ microglia cultures (*n* = 5/group). **(G–N)** Protein concentrations of interleukin (IL)-6 **(G)**, tumor necrosis factor (TNF)-α **(H)**, IL-1α **(I)**, IL-1β **(J)**, IL-10 **(K)**, monocyte chemoattractant protein (MCP-1, **L**), CXCL-1 **(M)**, CXCL-2 **(N)** in supernatants of LPS-stimulated CD11b+ microglia cultures (*n* = 5/group). HICT induced reduction in the total number of CD11b+ microglia **(B)** and in iNOS mRNA level in CD11b+ microglia **(C)** on the day of isolation. Training did not affect Arg-1 and CD206 **(C)**, BDNF and NGF mRNA levels **(D)**, ROS **(E)**, and NO **(F)** formation, and cytokine and chemokine secretion **(G–N)** in CD11b+ microglia in response to LPS stimulation *in vitro*. Data are mean ± SE. **(C–F)** Relative expression to SED group = 1. *p < 0.05, ** *p* < 0.01.

**Figure 6:**
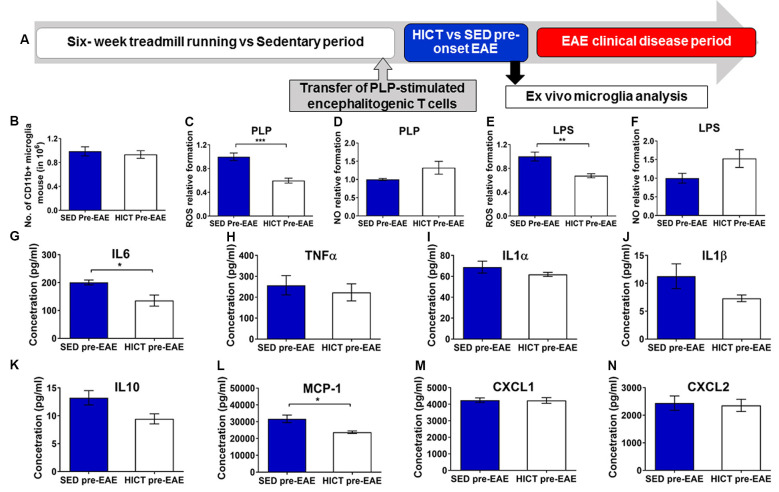
Reduced cytotoxic and proinflammatory properties in trained- derived microglia following the transfer of encephalitogenic T cells. **(A)** Mice were trained for 6 weeks. Seventy two hours before the last exercise bout, encephalitogenic T cells were injected into trained or sedentary control recipient mice. In general, mice develop clinical signs of experimental autoimmune encephalomyelitis (EAE) at 7–10 days post transfer of encephalitogenic T cells. Five days post-transfer (48 h after the last exercise bout), before the clinical onset of EAE, PLP-reactive CD11b+ microglia were isolated from brains of high-intensity continuous trained (HICT pre-EAE) or sedentary control (SED pre-EAE) mice. Isolated CD11b+ microglia were counted at the day of isolation (**B**; *n* = 8/group) and analyzed *ex vivo* after 24 h in culture with PLP (**C,D,G–N**; *n* = 5/group) or lipopolysaccharide (LPS, **E–F**; *n* = 5/group) stimulation. Levels of reactive oxygen species (ROS; **C,E**) and nitric oxide (NO; **D,F**) in supernatants of PLP- (**C,D**, respectively) or LPS- (**E,F**, respectively) stimulated CD11b+ microglia cultures (*n* = 5/group). **G–N** Protein concentrations of interleukin (IL)-6 **(G)**, tumor necrosis factor (TNF)-α **(H)**, IL-1α **(I)**, IL-1β **(J)**, IL-10 **(K)**, monocyte chemoattractant protein (MCP-1, **L**), CXCL-1 **(M)**, CXCL-2 **(N)** in supernatants of CD11b+ microglia cultures stimulated with PLP (*n* = 5/group). Training, followed by PLP-reactive encephalitogenic T cells transfer, induced reduction in ROS formation by CD11+ microglia in response to PLP **(C)** and LPS **(E)** stimulation *in vitro*; and in pro-inflammatory IL-6 cytokine **(G)** and MCP-1 chemokine **(L)** secretion after 24 h of PLP stimulation in culture. Training did not affect the total number of CD11b+ microglia on day of isolation **(B)**, the formation of NO in response to PLP **(D)** or LPS **(F)** stimulation *in vitro*, nor the secretion of TNF-α **(H)**, IL-1α **(I)**, IL-1β **(J)**, IL-10 **(K)**, monocyte chemoattractant protein (MCP-1, **L**), CXCL-1 **(M)**, CXCL-2 **(N)** in supernatants of **(J)**, IL-10 **(K)**, CXCL-1 **(M)** and CXCL-2 **(N)** after 24 h of PLP in culture. Data are mean ± SE. **(C–F)** Relative expression to SED group = 1. *p < 0.05, **p < 0.01, ***p < 0.001.

### Treadmill Exercise Training

Of the various exercise protocols that we have employed to date [MICT, HICT, and high-intensity interval training (HIIT)], HICT was found to be most effective in attenuating disease progression via systemic immunomodulation (Fainstein et al., 2019). Therefore, healthy mice underwent 6-weeks of treadmill running on a 5-lane treadmill designed for mice (Panlab Harvard Apparatus, USA), using the HICT protocol we established earlier (Einstein et al., 2018; Fainstein et al., 2019; see below, Table 1). The running speed in the HICT protocol was based on exhaustion speed performance tests, as previously described (Fainstein et al., 2019; see below).

**Table 1:** High-intensity continuous training (HICT) protocol.

	1st week	2nd week	3rd week	4th week	5th week	6th week
Duration per session	10 min	20 min	30 min	23 min		
				
Speed per session	23 cm/s			28 cm/s	30 cm/s	
Sessions per week	5 d/w			3 d/w		

*Min, minutes; cm/s, centimeters per second; d/w—days per week*.

### Exhaustion Speed Performance Test

Maximal running speed was assessed first by running the mice for 8 cm/s and then increasing the speed by 2 cm/s per minute until exhaustion. Exhaustion was defined by an inability/refusal to continue when encouraged with a bottle brush or a small puff of air. We defined HICT as 70–75% of exhaustion speed.

### Exercise Training Protocol

The 6 weeks of training started with 3 weeks of a preparatory period (Table 1). Each training session consisted of a 5-min warm-up at 8 cm/s. For the first, second, and third weeks, the warm-up was followed by 10, 20, and 30 min of training at 23 cm/min, respectively. In the subsequent 3 weeks, the trained mice were subjected to an incremental training protocol, reaching 23 min of training at 30 cm/min in the last 2 weeks. Based on the baseline exhaustion speed performance test, this training speed corresponds to an exercise intensity of 70–75% of maximal speed.

### Transfer Experimental Autoimmune Encephalomyelitis (EAE)

The PLP 139–151 transfer EAE model in mice was utilized as previously described (Einstein et al., 2007). EAE was induced in recipient HICT (*n* = 10) and SED (*n* = 10) mice by a transfer of encephalitogenic lymph node (LN)-T cells obtained from PLP-immunized donor mice (Figure 1A). Recipient EAE mice developed clinical signs of EAE 7–10 days post transfer of encephalitogenic T cells and were assessed daily for neurological symptoms for up to 50 days after EAE induction as follows: 0-asymptomatic; 1-partial loss of tail tonicity; 2-atonic tail; 3-hind leg weakness, difficulty to roll over, or both; 4-hind-leg paralysis; 5-4-leg paralysis; 6-death due to EAE (Einstein et al., 2018; Fainstein et al., 2019).

To assess disease severity, the maximal clinical score and the cumulative burden of disease (“area under curve”) were calculated for each mouse at the end of the follow-up. The burden of disease was calculated in individual mice by summation of the daily clinical scores along the entire follow-up period.

### Histopathology Analyses

At 12 days (at the acute phase of the disease) and 50 days (at the chronic phase of the disease) after encephalitogenic LN-T cell transfer, groups of SED (*n* = 6 for each time point) and HICT (*n* = 6 for each time point) EAE mice were sacrificed for histopathological analysis as previously described (Einstein et al., 2018). Serial paraffin-embedded transverse sections were obtained from mid-cervical, mid-thoracic, and mid-lumbar levels of the spinal cords. Sections were stained with hematoxylin and eosin (H&E), Luxol fast blue (LFB)/nuclear fast red, and Bielschowsky silver impregnation, to assess inflammation, demyelination, and axonal damage, respectively. Immunohistochemistry was performed in adjacent serial sections for macrophages (rat anti-mouse Mac3, 553322, 1:800, BD Pharmingen), T cells (monoclonal rabbit anti–CD3, RM-9107-SO; 1:800, Thermo-Scientific), and amyloid precursor protein (APP; monoclonal mouse anti-APP, MAB 348; 1:2,000; Millipore). Goat anti-rat (sc2041 Santa Cruz), goat anti-rabbit (BA 1000, Vector), and goat anti-mouse (BA9200, Vector) were used as secondary antibodies appropriately. The 3,3′-diaminobenzidine tetrahydrochloride was used as chromogen and sections were counterstained with hematoxylin. Immunofluorescence was performed for Iba-1 (polyclonal rabbit anti-IBA1, 019-19741, Wako), inducible nitric oxide (NO) synthase (iNOS; monoclonal mouse, sc-7271, Santa Cruz), arginase-1 (Arg-1 (polyclonal goat, sc-18354, Santa Cruz). Anti-rabbit CF488A (20012, Biotium), anti-goat CF555 (20039, Biotium) and anti-mouse CF555 (20030, Biotium) were used as secondary antibodies appropriately. Sections were mounted with Dapi (23004, Biotium). For each staining, the whole white matter of three sections per mouse was quantified, one section per spinal cord level. The number of immune cells in perivascular infiltrates were counted in H&E stained sections and reported as the total average number per square millimeter. Mac3+, CD3+ cells, and double-positive Iba-1+, iNOS+ and Iba1+, Arg-1+ were counted in the perivascular infiltrates and parenchyma, and reported as the total average number of each cell type per square millimeter. Demyelination was assessed by calculating the area of LFB loss. For the quantification of acute axonal injury, APP+ axonal swellings and spheroids were counted, and the average of APP+ profiles per square millimeter was calculated. For chronic axonal damage, the area of reduced axonal density in Bielschowsky silver staining was assessed. All pathology measurements were performed by using the ImageJ software analysis (ver. 1.51H, NIH, USA).

### *Ex vivo* Analyses of CD11b+ Microglia

Microglia were isolated (see below) from HICT and SED mice brain 48 h after the last exercise bout (*n* = 5–8/group). Microglia were isolated from healthy mice (Figure 4A) or 5 days following the transfer of encephalitogenic PLP-reactive T cells (transfer occurred 72 h before the last bout of exercise), before the appearance of clinical symptoms (pre-onset EAE mice, Figure 5A). This time point was selected according to our preliminary results confirming that no inflammatory infiltrates were present in the brain tissue (data not shown). Freshly isolated CD11b+ microglia were seeded on poly-L-lysine–covered 96 well plates and were activated over-night with either 200 ng/ml lipopolysaccharide (LPS, E. coli O111:B4, Sigma–Aldrich; for healthy-derived microglia) or 10 μg/ml PLP peptide (BioBasic; for pre-onset EAE- derived microglia). Activated microglia were analyzed for reactive oxygen species (ROS) and nitric oxide (NO) formation [(using enzyme-linked immunosorbent assay (ELISA)], cytokine gene determination [real time-PCR (RT-PCR)] and cytokine and chemokine secretion (MAGPIX System).

### Microglia Isolation

Brain tissue from HICT and SED healthy or pre-onset EAE mice were dissociated to single-cell suspension, using the Neural Tissue Dissociation Kit (Miltenyi Biotec). Myelin was removed using Percoll (GE Healthcare) followed by microglia isolation using CD11b microbeads and MS columns (Miltenyi Biotec) according to manufacturer instructions. The degree of microglia enrichment was assessed by CD11b (BD Bioscience, M1/70) staining and flow cytometry analysis (Beckman Coulter). In all experiments at least 85% of isolated cells expressed CD11b.

### Nitric Oxide (NO) and Reactive Oxygen Species (ROS) Formation

NO formation was assessed (on 25 × 10^6^ cells/well) using Greiss Reagent System according to manufacturer’s protocol (Promega, G2930) and quantified using an ELISA plate reader (Tecan Spark 10M). ROS formation was measured (on 5 × 10^6^ cells/ well) using DCFDA dye according to the manufacturer’s protocol (Abcam, AB-ab113851) and quantified with an ELISA plate reader (Beckman Coulter DTX 880 multimode detector).

### Cytokine Gene Determination

Total RNA was prepared using the RNeasy Plus Mini Kit (QIAGEN) from microglia. cDNA was prepared from 300 ng total RNA using qScript cDNA Synthesis Kit (Quanta Biosciences). The reaction mixture included 1 μl of cDNA, 300 nm of appropriate forward and reverse primers (Agentek), and 5 μl PerfeCTA SYBR Green FastMix ROX (Quanta Biosciences) to a total volume of 10 μl. Gene amplification was carried out using the StepOnePlus real-time PCR system (Applied Biosystems).

### Cytokine and Chemokine Secretion Assessment

Cytokine and chemokine concentrations in LPS- or PLP-stimulated microglia supernatants were measured with MILLIPLEX^®^MAP mouse high sensitivity magnetic bead panels, according to manufacturer instructions (EMD Millipore Corp., Burlington, MA, USA). The Luminex xMAP^®^ technology was utilized, based on immunoassay performed on the surface of fluorescent-coded magnetic beads MagPlex^®^-C microspheres. Acquisition and data analysis were performed using Luminex analyzer (MAGPIX^®^) software (Bio-Rad Laboratories, Hercules, CA, USA).

### Statistical Analyses

The normality of the distribution of variables was tested by the Shapiro–Wilk test followed by the appropriate statistical test for comparison. For performance tests, the values before and after training for each experimental group were compared using the Student’s paired* t-*test. For clinical disease severity parameters (i.e., day of onset, maximal clinical score, and burden of disease), pathology parameters, and microglia analyses, experimental groups were compared using the unpaired Student’s *t*-test or the two-tailed Mann-Whitney test, according to the normality test. Data were analyzed in GraphPad Prism software v.5. Differences were considered statistically significant at *p* < 0.05. All data are presented as mean ± standard error of the mean (SE).

## Results

### High-Intensity Continuous Training (HICT) Improves Physical Performance

Based on performance test conducted before and at the end of the training program (*n* = 7), HICT significantly improved both maximal speed (11%, 42.6 ± 0.6 vs. 47.1 ± 0.6 cm/s, *p* < 0.05) and exercise tolerance (93%, 15:09 ± 0:20 vs. 29:39 ± 2:08 min:s, *p* < 0.001). No significant performance changes were noted in SED mice (*n* = 7; exhaustion speed: 42.0 ± 1.2 vs. 42.0 ± 1.3 cm/s, *p* > 0.05; exercise tolerance: 14:25 ± 1:10 vs., 17: ± 1:32 min:s, *p* > 0.05).

### Training Induces Direct CNS Protection From Autoreactive Encephalitogenic T Cells

The transfer of PLP-reactive encephalitogenic T cells from donor mice induced a milder clinical course of EAE in HICT recipient mice, as compared to SED mice (Figures 1B,C). The day of onset was significantly delayed by 3 days in the HICT group (*p* < 0.01) vs. SED group and the overall burden of disease was significantly lower (43%, *p* < 0.05) in HICT mice than in control SED mice (Figure 1C). Although the average maximal clinical score of disease in the HICT group was decreased by 17%, the decrease was not statistically significant (*p* > 0.05, Figure 1C).

Demyelination and acute axonal injury were assessed at the peak of the acute relapse (day 12 post-transfer, Figures 2A–F). LFB staining showed a significant 37% reduction in the extent of demyelination in HICT EAE mice vs. control SED EAE mice (*p* < 0.001, Figures 2A–C). APP immunohistochemistry at the acute phase showed a 32% reduction in the number of injured axons (*p* < 0.05, Figures 2D–F). Demyelination and chronic axonal loss were evaluated also at the chronic phase of EAE (day 50 post-transfer, Figures 2G–L). At this stage, the protective effect of HICT was even more pronounced. In HICT EAE mice the area of demyelination (Figures 2G–I) and chronic axonal loss (Figures 2J–L) were reduced by 49% and 48%, respectively (*p* < 0.001).

Since the severity of tissue damage in EAE is related to the autoimmune inflammatory process (Martin and Mcfarland, 1995; Bitsch et al., 2000; Linker et al., 2005), we examined whether HICT protected the CNS from the destructive inflammatory process. SED EAE mice exhibited extensive acute inflammation (Figures 3A,D,G), whereas HICT EAE mice exhibited substantially less inflammation (Figures 3B,E,H). The overall immune cell, CD3+ T- cell and Mac3+ macrophages infiltrations were decreased by 21%, 43% and 50%, respectively (*p* < 0.001, Figures 3C,F,I). Evaluation of neuroinflammation at the chronic phase of the disease (day 50 post-transfer, Figures 3J–R) revealed no significant differences in inflammatory cell counts between the two experimental groups (*p* > 0.05).

### Training Modulates Microglial Inflammatory and Neurotoxic Properties

The *in vivo* experiments showed that HICT induced CNS protection against the PLP-reactive encephalitogenic T cells and their destructive effects on the CNS. Since training protected the CNS directly, we examined the effect of HICT on the CNS innate immune system. Microglia mediate the recruitment and activation of systemic immune cells and induce neurotoxicity in chronic neuroinflammatory and neurodegenerative disorders (Heneka et al., 2014). We, therefore, examined the microglial profile *in vivo* at the acute phase of EAE. HICT induced a marked 61% decrease in the number of neurotoxic Iba1+, iNOS+ (M1 type) microglia compared to SED control (*p* < 0.05, Figures 4A–C). No differences were noted in the number of M2 type Iba-1+, Arg-1+ microglia between HICT and SED EAE mice (*p* > 0.05, Figures 4D–F).

Since systemically administered PLP-reactive encephalitogenic T cells encountered the CNS milieu that had already been modulated by exercise training we next characterized microglia at the end of the training or sedentary period, before the transfer of encephalitogenic T cells. To that end, CD11b+ microglia were isolated for *ex vivo* evaluation (Figure 5A). The total number of CD11b+ microglia was significantly reduced by HICT vs. SED controls (33%, *p* < 0.01, Figure 5B). Moreover, HICT decreased the mRNA level of iNOS (46%, *p* < 0.05, Figure 5C) in isolated CD11b+ microglia, but did not affect mRNA levels of Arg1 and CD206 (*p* < 0.05, Figure 5C), nor brain-derived neurotrophic factor (BDNF) or nerve growth factor (NGF; *p* > 0.05, Figure 5D). Also, the ability of CD11b+ microglia from HICT mice to respond to LPS stimulation *ex vivo* was not affected when compared to SED mice, in terms of reactive oxygen species (ROS; *p* > 0.05, Figure 5E) and nitric oxide (NO; *0* > 0.05, Figure 5F) formation, as well as the release of cytokines (*0* > 0.05, Figures 5G–K) and chemokines (*p* > 0.05, Figures 5L–N) These findings indicate that HICT did not alter the ability of microglia to respond to an infectious challenge.

Then the response of brain microglia to the transfer of encephalitogenic T cells was examined at 5 days post-transfer, a time point before the initial invasion of autoimmune cells into the CNS, and before any clinical manifestations (Figure 6A). Noteworthy, induction of EAE by T-cell transfer in HICT mice restored brain content of microglia to control levels (Figure 6B), indicating normal reactivity. However, when re-stimulated with PLP in culture, microglia derived from HICT mice exhibited a marked reduction in ROS formation compared to SED mice (39%, *p* < 0.001, Figure 6C). Reduction in ROS formation was also observed when HICT- derived microglia were stimulated with LPS in culture, compared to control (33%, *p* < 0.01, Figure 6E). HICT did not affect NO formation by microglia after PLP (*p* > 0.05, Figure 6D) or LPS (*p* > 0.05, Figure 6F) stimulation *in vitro*, compared to SED controls. Finally, we examined the effect of training on the inflammatory profile of microglia isolated from the brain at 5 days after the transfer of encephalitogenic T cells and following PLP re-stimulation* in vitro*, as reflected by measuring cytokine and chemokine secretion (Figures 6G–N). HICT mice-derived microglia exhibited reduced interleukin (IL)-6 (32%, *p* < 0.05, Figure 6G) and monocyte chemoattractant protein (MCP)-1 (25%, *p* < 0.05, Figure 6L) release. There was no difference in the release of other cytokines (*p* > 0.05, Figures 6H–K)/chemokines (*p* > 0.05, Figures 6M,N).

## Discussion

We investigated the direct effects of training on autoimmune neuroinflammation using the transfer EAE model. High-intensity training provided direct protection to the CNS from autoimmune neuroinflammation, resulting in attenuated disease progression, and reduction in inflammation-driven demyelination and axonal loss. The neuroprotective effect appears to be mediated, at least partly, by modulating the CNS innate immune system and reducing the microglial neurotoxic and pro-inflammatory response to T-cell mediated autoimmune neuroinflammation. Our study provides the first demonstration of the direct neuroprotective effect of training on autoimmune neuroinflammation and points to the critical role of training intensity in this process.

Earlier studies suggested various mechanisms to account for the beneficial effects of training on EAE (Rossi et al., 2009; Bernardes et al., 2013; Patel and White, 2013; Benson et al., 2015; Pryor et al., 2015; Souza et al., 2016; Kim and Sung, 2017). However, these studies used active EAE models that could not distinguish between the effects of training on the systemic immune system vs. direct protective effects on the CNS to reduce encephalitogenicity. The transfer EAE model used in the present study enables this distinction.

In our previous work, we utilized the PLP-transfer EAE model to show that moderate-intensity training of recipient mice did not affect the clinical course nor the CNS pathology of EAE (Einstein et al., 2018). Here we show that when recipient mice undergo high-intensity training, their CNS is protected from the deleterious effects of encephalitogenic T cells, resulting in milder tissue pathology and clinical symptoms of EAE. Thus, training intensity is paramount for inducing direct neuroprotection against autoimmune neuroinflammation.

Interestingly, the inflammatory process was attenuated in HICT mice only during the acute phase. Since the training program ended soon after (72 h) injection of encephalitogenic T cells, the protection of training on the CNS inflammatory process may be a short-term effect. Alternatively, the lack of attenuation of neuroinflammation at the chronic phase reflects the observed partial spontaneous resolution of neuroinflammation after the acute relapse. However, most tissue injury occurs in the early phase of EAE and was therefore reduced in HICT mice in both the acute and chronic phases.

Microglia play a crucial role in the maintenance of CNS homeostasis in health and disease (Hammond et al., 2018). Activated microglia produce a wide range of inflammatory and toxic mediators, which contribute to the recruitment of immune cells and the spread of the inflammatory response in the CNS of MS and EAE (Raivich and Banati, 2004; Huizinga et al., 2012; Rissanen et al., 2018). The observation that a decrease in microglial reactivity has been associated with a beneficial effect in EAE (Heppner et al., 2005; Bhasin et al., 2007; Guo et al., 2007) led to the perception of microglia as detrimental cells in EAE pathogenesis. Thus, we hypothesized that microglia are potential key components to mediate the training-induced neuroprotection in the CNS. Indeed, HICT induced a substantial decrease in the number of iNOS+ microglia in EAE mice. Interestingly, HICT did not affect the number of Arg-1+ microglia. These findings suggest that training reduces the neurotoxic profile of microglia, rather than inducing their shift to M2 phenotype.

Earlier studies demonstrated that microglial reactivity precedes the onset of EAE and that inhibition of their activation suppresses the development and maintenance of inflammatory lesions in the CNS (Ponomarev et al., 2005). We therefore further examined the profile and characteristics of microglia *ex vivo* at the end of the training program and following EAE induction, but before infiltration of encephalitogenic T cells into the CNS. The current study suggests that HICT modulates microglia phenotype following EAE induction to attenuate neuroinflammation. In our experimental paradigm, training did not prevent the formation of encephalitogenic T cells, which were obtained from donor mice, but rather affected brain innate immune cells in trained recipient mice, and prevented neuroinflammation and tissue destruction.

Importantly, both over-activation and suppression to a mal-functional state of microglia are deleterious to brain health (Luo and Chen, 2012; Cherry et al., 2014). Restoration of microglial homeostasis, rather than their elimination or total inhibition, is the preferred therapeutic target. Accordingly, our findings in healthy mice demonstrate that training reduces the number of microglial cells (the extent to which apoptotic cells impact the cell count cannot be assessed with the methods employed in the present study), without affecting their ability to produce immune mediators, nor affecting their M1/M2 phenotype. Furthermore, training did not induce a general suppressive effect on microglia derived from mice before the onset of EAE. Rather, training-induced modulation of microglial neurotoxic phenotype, as indicated by reducing ROS, IL-6 cytokine, and MCP-1 chemokine production in response to PLP and LPS stimulation. The lack of a general suppressive effect of training on microglial function is important in enabling the still activated, HICT-derived microglia to participate in their homeostatic and regenerative roles.

A key finding in the current study was the reduction in PLP-mediated secretion of the pro-inflammatory cytokine IL-6 and the MCP-1 chemokine and ROS formation by HICT pre-onset EAE microglia. Intrinsic production of IL-6 in the brain is necessary for the induction of EAE and plays an important role in modulating the development and progression of the disease (Mendel et al., 1998; Lock et al., 2002; Quintana et al., 2009). IL-6 is necessary for the induction of cerebrovascular adhesion molecules, which are crucial for leukocyte trafficking to the CNS in EAE (Eugster et al., 1998). MCP-1 is detected before the onset of inflammation in EAE and plays an important role in the influx of inflammatory cells into the CNS (Carr et al., 1994; Berman et al., 1996; Juedes et al., 2000). There is a positive correlation between the expression of MCP-1 in the CNS and the degree of CNS inflammation and severity of EAE (Mahad and Ransohoff, 2003). We observed a significant reduction in immune cell infiltrates in the CNS of the trained group compared to the sedentary group at the acute phase of EAE. This can be attributed to reduced release of chemotactic factors (such as MCP-1) by microglia in the trained group, which is consistent with the idea that training reduces microglial- driven pathogenesis of neuroinflammation. Taken together, our finding suggests that the inhibitory effect of HICT on IL-6 and MCP-1 secretion by microglia contribute to the decrease in inflammatory infiltration into the CNS and attenuation of acute CNS inflammation, tissue injury, and clinical severity of EAE.

The positive effects of HICT on disease progression in the present study is not associated with a decrease in microglial NO formation in response to PLP stimulation. In contrast, the ROS response to PLP was markedly decreased by HICT. NO and ROS play a central role in microglial neurotoxicity (Heneka et al., 2014). Indeed, excessive formation of ROS contributes to the death of neurons of the mammalian CNS in various neurodegenerative diseases (Martindale and Holbrook, 2002; Uttara et al., 2009). Accordingly, ROS have been implicated as a mediator of demyelination and axonal injury in both EAE and MS (van der Goes et al., 1998; Gilgun-Sherki et al., 2004). The formation of ROS is dependent on the simultaneous production (starting with the generation of the superoxide anion (${\text{O}}_{{\text{2}}^{-}}^{*}$), which leads to the formation of other ROS species, such as H_2_O_2_ and hydroxyl (radicals) and removal of ROS [generally achieved by antioxidant enzymes such as superoxide dismutase, glutathione peroxidase (using reduced glutathione, GSH) and catalase; Powers and Jackson, 2008]. Exercise results in increased production of ROS, especially at high intensities (Zhang et al., 2007; Powers and Jackson, 2008), and it is, therefore, unlikely that the decreased formation of ROS following HICT is due to a decreased capacity to produce ROS. On the other hand, it is well documented that exercise training increases the activity of antioxidant enzymes and the levels of GSH in skeletal muscle (Ji, 2008). Similarly, exercise training also increases antioxidant defense systems in some but not all rodent brain cell populations (Freitas et al., 2019; Li et al., 2019; Song et al., 2020). Therefore, we hypothesize that the decreased ROS response to PLP-stimulation in HICT microglia derives from increased removal of ROS species owing to elevated antioxidant defense systems (Vilhardt et al., 2017). If this hypothesis is correct, then one would expect that administration of exogenous antioxidants to EAE-SED mice would mimic the effects of HICT on disease progression. Indeed, a recent study using experimental conditions comparable to those of the current study demonstrated that administration of a mitochondria-targeted antioxidant (Mito-Q) to EAE-SED mice diminished the clinical progression of the disease (Mao et al., 2013). Moreover, the increased mRNA levels of CD11b and IL6 in spinal cords of EAE-SED mice were markedly decreased by administration of Mito-Q. Additionally, neurodegeneration and neuroinflammation in spinal cord sections of EAE-SED mice were also diminished by Mito-Q. Taken together, our results, together with those from the literature, suggest that HICT attenuates disease progression by inhibiting ROS formation in microglia. Although the emphasis of microglia in the mediation of the beneficial effects of exercise on neuroprotection is supported by considerable experimental evidence (Mee-Inta et al., 2019), our findings do not preclude a positive effect of exercise training on other brain cell populations as well.

In conclusion, high-intensity training induces a direct neuroprotective effect in an experimental model of autoimmune neuroinflammation. CNS microglia serve as a key therapeutic target for neuroinflammation, and their modulation by HICT may reduce their neurotoxic and pro-inflammatory properties. Elucidating the mechanisms underlying the positive effects of exercise training on autoimmune neuroinflammation will facilitate the translation of basic research findings to clinical benefits for neurological patients. Importantly, our experimental design here illustrates the preventive role of training on the development of EAE. Thus, an active lifestyle and regular exercise training can modulate the brain immune cells to optimally respond to deleterious autoimmune encephalitogenic cells. These findings may also apply to other neurodegenerative diseases driven by microglial neurotoxicities, such as AD and PD.

## Data Availability Statement

The raw data supporting the conclusions of this article will be made available by the authors, without undue reservation.

## Ethics Statement

The animal study was reviewed and approved by Institutional animal care and use committee, Ariel University.

## Author Contributions

YZ designed research studies, conducted experiments, acquired data, analyzed data, and wrote the first draft. NF designed research studies, conducted experiments, acquired and analyzed data. OT conducted experiments, acquired and analyzed data. YG, LH, SS, HN, and SZ conducted experiments. NG and AK analyzed data and critically reviewed the manuscript. TB-H designed the research studies, analyzed data, and critically reviewed the manuscript. OE designed research studies, analyzed data, and wrote the manuscript. All authors read and approved the final manuscript.

## Conflict of Interest

The authors declare that the research was conducted in the absence of any commercial or financial relationships that could be construed as a potential conflict of interest.
